# Prenatal ultrasound diagnosis, associated anomalies and pregnancy outcomes of fetal right aortic arch

**DOI:** 10.3389/fcvm.2025.1521338

**Published:** 2025-01-29

**Authors:** Yuting Xie, Zongjie Weng, Ronghua Wang, Qiumei Wu, Wen Ling, Jinwen Chen, Shan Guo

**Affiliations:** ^1^ Department of Medical Ultrasonics, Fujian Maternity and Child Health Hospital, College of Clinical Medicine for Obstetrics & Gynecology and Pediatrics, Fujian Medical University, Fuzhou, China; ^2^ Department of Medical Ultrasonics, Second Hospital of Sanming City, Sanming, China

**Keywords:** fetus, echocardiography, right aortic arch, prenatal diagnosis, pregnancy outcome

## Abstract

**Objective:**

The aim was to summarise the prenatal ultrasound characteristics, associated anomalies and pregnancy outcomes of fetal right aortic arch (RAA) and to discuss the value of prenatal ultrasound diagnosis and prognostic analysis.

**Methods:**

We retrospectively analysed 157 cases of fetal RAA diagnosed via prenatal ultrasound in our hospital from October 2017 to October 2022. RAA features were characterised by comparing prenatal ultrasound data with anatomical casting results after pregnancy termination or postnatal imaging and surgical intervention to analyse the prognosis and misdiagnoses of fetal RAA.

**Results:**

Of the 157 fetal RAA cases, 50 (31.8%) cases were isolated RAA and 107 (68.2%) cases were nonisolated RAA. In terms of typing, 78 cases (49.7%) of right aortic arch-aberrant left subclavian artery (RAA-ALSA) and 75 cases (47.8%) of right aortic arch-mirror branch (RAA-MB), 3 cases (1.9%) of right aortic arch-isolated left subclavian artery (RAA-ILSA) and 1 case (0.6%) of right aortic arch-isolated left innominate artery (RAA-ILINA) were identified, and the incidence of combined cardiac anomalies and chromosomal anomalies was significantly greater in the RAA-MB group than the RAA-ALSA group. The live birth rate was significantly lower in the nonisolated RAA group than the isolated RAA group, and the prognosis of RAA-MB was significantly worse than that of RAA-ALSA. Among the 76 surviving patients, 72 (94.7%) cases were correctly diagnosed via prenatal ultrasound, and 4 (5.3%) cases had missed diagnoses and misdiagnoses. Of the 81 terminated pregnancies, 19 cases received pathological anatomy or vascular casting, including 18 cases with results consistent with the prenatal ultrasound and 1 case with inconsistent results.

**Conclusions:**

Prenatal echocardiography is useful for diagnosing fetal RAA. It is also necessary to classify RAA types as accurately as possible and detect the presence of potential cardiac and extracardiac anomalies and genetic abnormalities, which facilitates prenatal counselling and prognostic assessment of fetuses with RAA.

## Introduction

Right aortic arch (RAA) is a congenital vascular malformation that manifests as a transverse aortic arch on the right side of the trachea (1). The prevalence of RAA is approximately 0.1% (2), and RAA may be independent or combined with other malformations (3). Complete or incomplete vascular rings may form on the basis of the branching of the aortic arch and the ductus arteriosus (DA) (4). The clinical presentation and prognosis of RAA vary greatly, and most cases of RAA without complex malformations have a good prognosis, whereas severe cardiac and extracardiac malformations and chromosomal abnormalities are associated with a poor prognosis (5, 6). Therefore, it is particularly important to accurately identify and diagnose fetal RAA prenatally and to determine whether RAA is associated with other structural and genetic anomalies, which is highly important for clinical decision-making and prognostic assessment.

Prenatal ultrasound is an important means of diagnosing fetal RAA, providing an assessment of fetal cardiac macrovascular imaging by fetal echocardiography through a variety of views (7). Several studies on fetal RAA have been performed. Lacunza (8) proposed that descending aortic arch coronary views, three-vessel-tracheal views and long-axis views of the aorta could be used to diagnose fetal RAA. Yu et al. (9) applied two-dimensional and spatiotemporal image correlation (STIC) to identify the branching patterns of RAA. However, owing to the small internal diameters of the fetal aortic arch branches and the limitations of ultrasound resolution, the scanning section, the sampling angle and flow sensitivity, it is difficult to determine the complete origins and course of some branches (10), which makes accurate diagnosis and differentiation of RAA challenging. In addition, several studies have shown that fetal RAA is closely associated with chromosomal abnormalities (11), especially 22q11 microdeletion (12). Maya et al. (13) reported that microdeletion microrepetitions with abnormal copy numbers were detected in 6.4% of fetuses with RAA via chromosomal microarray analysis (CMA). However, little is known about the differences in chromosomal anomalies among the various types of RAA.

On the basis of the previous background and the accumulated working experience of our centre over the years, this study summarised the prenatal ultrasound features and combined abnormalities of RAA and its subtypes and analysed pregnancy outcomes and prognoses, which aims to improve the accuracy of prenatal diagnosis and provide a theoretical basis for clinical decision-making, and prognostic assessment of fetal RAA.

## Methods

This retrospective study analysed 81,431 fetuses diagnosed via prenatal ultrasound from October 2017 to October 2022. There were 157 fetuses with RAA diagnosed during the study. The pregnancy age ranging from 24 to 41 years, and the mean gestational age at RAA diagnosis was 22.85 ± 5.68 weeks. Informed consent forms were obtained from the guardians of all the study subjects. The study was approved by the Ethics Committee of Fujian Maternity and Child Health Hospital, Fujian Medical University (2024KY175-02). Data on prenatal diagnosis and postnatal follow-ups were obtained from the electronic medical records. The prenatal ultrasound results were compared with the findings of postnatal imaging, surgical results, pathological anatomy, or casting.

### Fetal echocardiography

All patients underwent ultrasound screening performed with GE Voluson E8 or E10, Siemens ACUSON S2000, Philips IE33 ultrasound machine with a 4.0–8.0 MHz probe. The ultrasound settings for fetal echocardiography and pregnancy examination were set according to the parameters recommended by the manufacturer.

According to the International Society of Ultrasound in Obstetrics and Gynecology (ISUOG) fetal cardiac screening guidelines (14), the fetal heart was scanned via segmental sequence analysis. For suspected RAA cases, three-vessel series sections (three-vessel sections, trivascular-tracheal sections, trivascular-pulmonary artery bifurcation sections), long-axis aortic sections and aortic coronal sections were used for observation, and the left/right ventricular outflow tract, ascending aorta, aortic branches, DA were explored. The three-step approach for echocardiography was as follows: (1) clarify the laterality, number and horizontal position of the aortic arch and the position of the beginning section of the descending aorta; (2) identify the laterality number and connection mode of the DA; and (3) trace the issuance and travel of the aortic arch branches and pulmonary artery branches and their spatial position in relation to the trachea. In fetuses with RAA detected by ultrasound, careful examination was performed to determine the presence of other intra- or extracardiac anomalies.

### Pathological anatomy and vascular casting

The pathological anatomy of the specimens was combined with *in situ* observations and ex vivo immobilisation. The specific steps were as follows: (1) After the removal of the thymus, the heart and large blood vessels were exposed, and the pericardium was removed; (2) *in situ* observation of the heart was performed, with a focus on (a) the position, axis, size, and shape of the heart; (b) the external shape of the atria and ventricles; (c) the location and size of the aorta; and (d) the size and continuity of the aortic arch, ductus arteriosus, and descending aorta after the displacement of the lungs; (3) the heart–lung tissue was removed and fixed with formalin liquid; and (4) the fixed heart specimen was autopsied along the direction of blood flow to reveal the atrium, ventricle, atrioventricular septum, and valve and the opening position, inner diameter, branched blood vessel, and direction of the aorta. The samples were photographed and archived before and after casting, and the casting results were recorded.

### Neonatal echocardiography

Postnatal echocardiography was performed with a Philips EPIQ 7C and IE Elite ultrasound diagnostic instrument, with the probe frequency set at 3.0–8.0 MHz. In accordance with the American Society of Echocardiography (ASE) paediatric echocardiography guidelines (15), the heart was comprehensively scanned via segmental analysis to observe the origin, internal diameter, and blood flow of the pulmonary artery and its branches. The development of other cardiovascular structures was also evaluated.

### Types of fetal RAA

The RAA was classified into four types according to the degree of aortic arch branching abnormalities: right aortic arch-aberrant left subclavian artery (RAA-ALSA), right aortic arch-mirror branch (RAA-MB), right aortic arch-isolated left subclavian artery (RAA-ILSA), and right aortic arch-isolated left innominate artery (RAA-ILINA).

### Definition of combined fetal RAA anomalies

RAA without combined anomalies was defined as isolated RAA, and RAA with combined abnormalities was defined as nonisolated RAA. The combined anomalies included soft makers, intracardiac abnormalities, and extracardiac abnormalities. The soft markers, frequently temporary anatomic findings, include thickened nuchal translucency, choroid plexus cysts, hypoplastic nasal bone, hyperechogenic bowel, pyelectasis, choroid ventriculomegaly, short femur, mild tricuspid regurgitation, echogenic intracardiac focus, single umbilical artery and so on.

### Statistical analysis

All the statistical analyses were performed via SPSS v25.0 software. The quantitative data are expressed as the means ± standard deviations and the counting data were expressed as frequency or percentage. Comparisons were performed via Student's *t*-tests, assuming unequal variance between groups, and Fisher's exact chi-square test. *P* < 0.05 was considered statistically significant.

## Results

**Figure 1:**
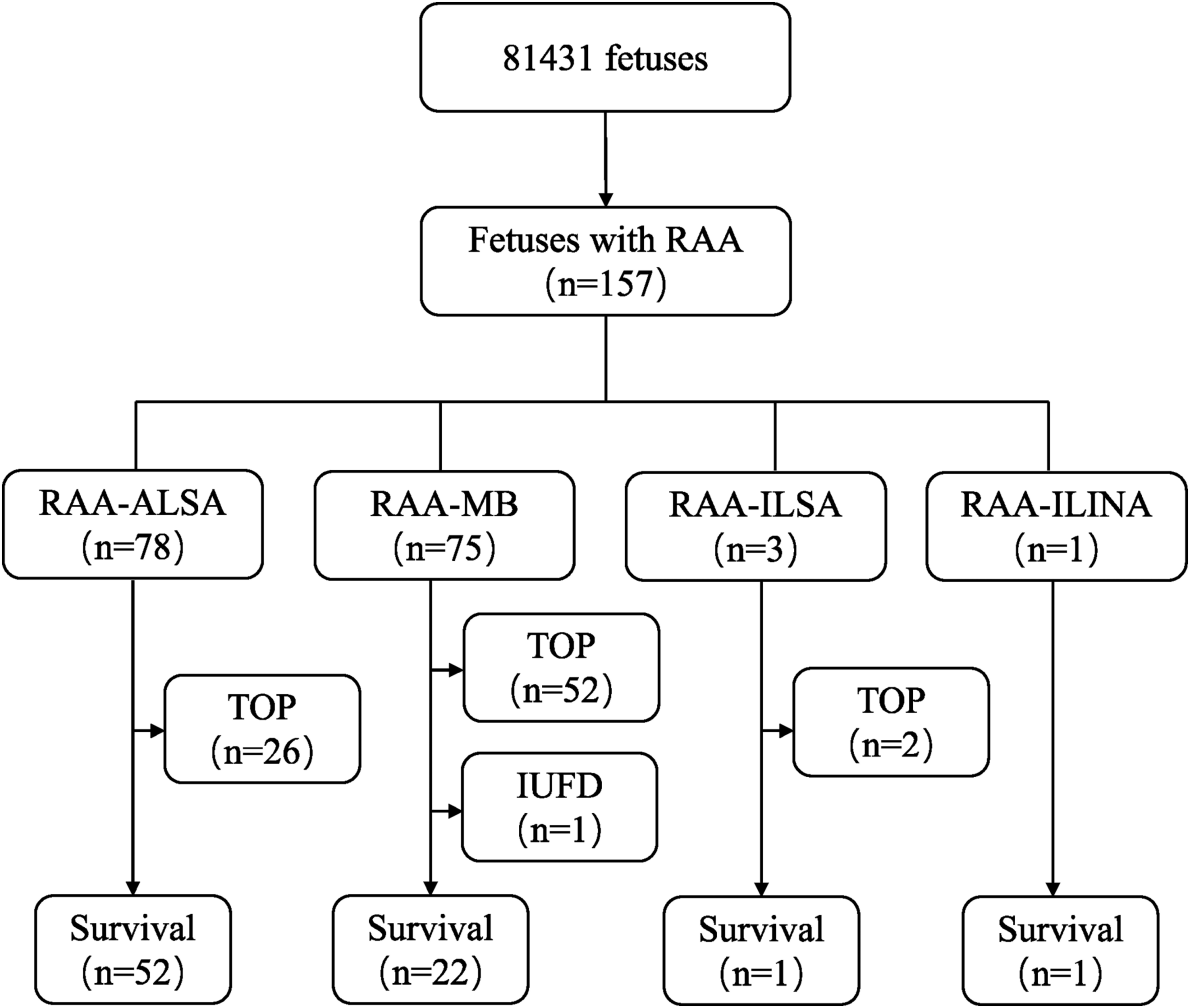
Flowchart of patients in this cohort with a prenatal diagnosis of RAA. RAA, right aortic arch; RAA-ALSA, right aortic arch-aberrant left subclavian artery; RAA-MB, right aortic arch-mirror branch; RAA-ILSA, right aortic arch-isolated left subclavian artery; RAA-ILINA, right aortic arch-isolated left innominate artery; TOP, termination of pregnancy; IUFD, intrauterine fetal demise.

The flowchart of this study is shown in Figure 1. Among the 157 fetuses with RAA, 76 were born alive and 1 experienced intrauterine fetal demise (IUFD). A total of 80 pregnancies were terminated. A total of 62 cases were performed by interventional fetal chromosome examinations. Among the 76 live-born infants, 72 cases (94.7%) were correctly diagnosed via prenatal ultrasound, and 4 cases (5.3%) were misdiagnosed. Of the 81 pregnancy terminations, 19 cases were comfirmed by pathological autopsy or vascular casting.

### Prenatal ultrasound findings of fetal RAA

**Figure 2:**
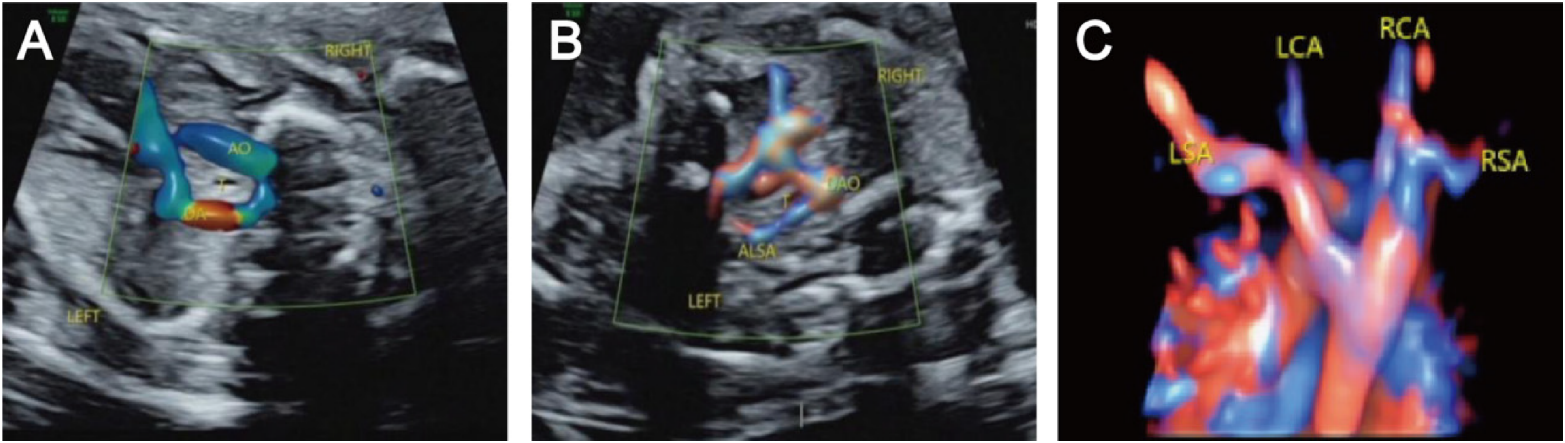
Right aortic arch with an aberrant left subclavian artery and left ductus arteriosus. **(A)** Three-vessel and trachea view showing that the aortic arch is located on the right side of the trachea, the ductus arteriosus and pulmonary artery are located on the left side of the trachea, and the “U” shaped blood vessels surround the trachea; **(B)** Bilateral subclavian artery view showing that the left subclavian artery starts from the beginning of the descending aorta, travelling obliquely towards the left arm at the back of the trachea, and forms a “C” shaped blood vessel with the aortic arch around the trachea; **(C)** STIC imaging showing that the four branches of the aortic arch are the left common carotid artery, right common carotid artery, right subclavian artery, aberrant left subclavian artery, and left subclavian artery starting from the beginning of the descending aorta and travelling left from the posterior trachea.

**Figure 3:**
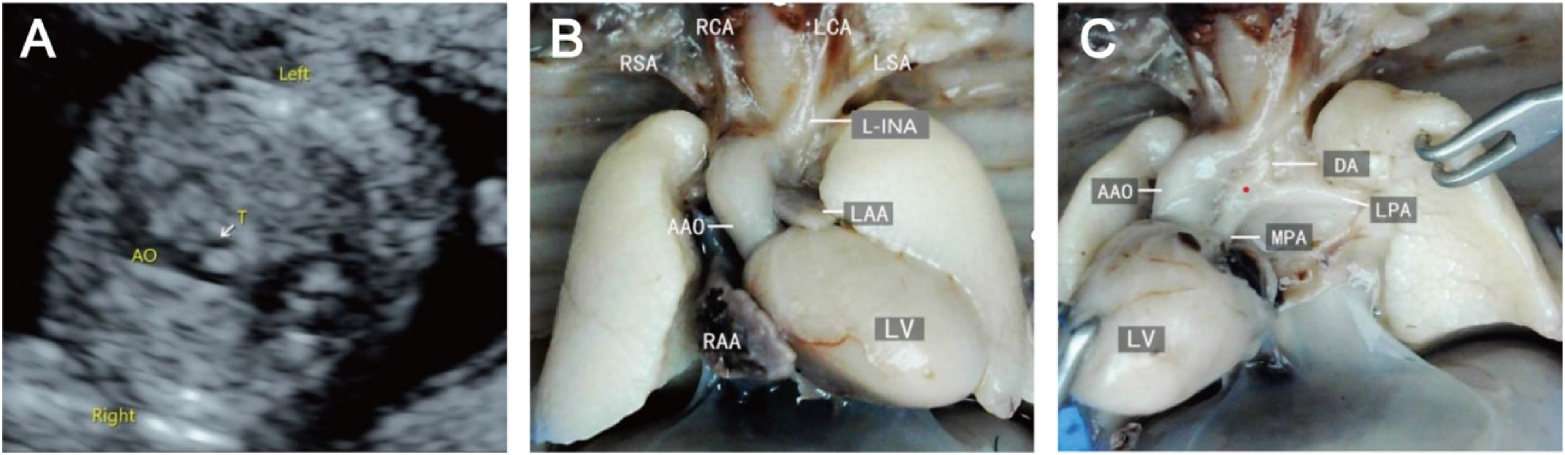
Right aortic arch with a mirror branch, right arterial catheter ultrasound and micropathological anatomical comparison. **(A)** Three-vessel and trachea view showing that the aortic arch is located on the right side of the trachea; **(B/C)** Labour induction specimens are shown in the right aortic arch with the right arterial catheter, and the aortic arch shows three branches from, near to far, the left innominate artery, right common carotid artery, and right subclavian artery.

**Figure 4:**
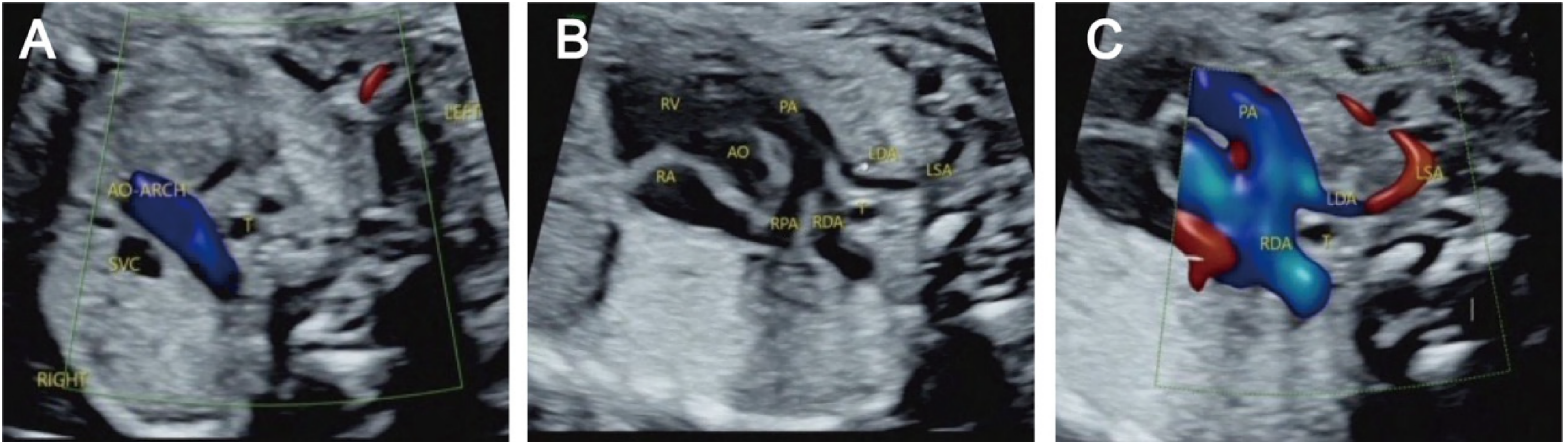
Right aortic arch with an isolated left subclavian artery and double ductus arteriosus. **(A)** Three-vessel and trachea view showing that the aortic arch is located on the right side of the trachea; **(B/C)** An arterial ductus is observed on each side of the trachea, the right arterial ductus is located on the right side of the trachea, and the left subclavian artery is connected to the aortopulmonary artery through the left ductus arteriosus.

The prenatal ultrasound feature of RAA is an aortic arch on the right side of the trachea, and depending on the different connections with the branches of the aortic arch and the DA, vascular rings may form. The type of fetuses with RAA and their lateral distribution of the DA are shown in Table 1. Imaging of RAA-ALSA revealed that the left subclavian artery originated from the beginning of the descending aorta, circled around the posterior part of the trachea, and travelled obliquely to the left side of the left axilla. Of the RAA-ALSA cases, 67 cases were accompanied by left ductus arteriosus (LDA), which formed a “U”-shaped vascular ring centred on the trachea by the RAA, LDA, and ALSA (Figure 2); 11 cases were accompanied by right ductus arteriosus (RDA) or absence of ductus arteriosus (ADA), which formed a “C”-shaped vascular ring by the RAA-ALSA. In 24 cases of RAA-MB with LDA, the RAA and LDA formed a “U”-shaped vascular ring around the trachea; in 17 cases, the LDA was connected to the beginning of the left innominate artery, and the catheter travelled vertically upwards and downwards; in 27 cases with RDA, the right side of the trachea where the transverse arch of the aorta met the ductus arteriosus showed a “V”-shaped vascular sign (Figure 3); in 1 case with double ductus arteriosus (DDA), an “O”-shaped vascular ring was formed; and in 6 cases, the DA was absent, and the echoes of the conduit were not detectable. All 3 fetuses with RAA-ILSA had a right aortic arch emanating from the left common carotid artery, the right common carotid artery, and the right subclavian artery from proximal to distal, with the left subclavian artery connecting to the pulmonary artery via the LDA; 2 of these patients had LDA, and 1 had DDA (Figure 4), with no vascular ring formation. In one case of RAA-ILINA with LDA, the aortic arch had only two branches, the right common carotid artery and the right subclavian artery, and the left innominate artery was connected to the pulmonary artery through a left-site DA, with no vascular ring formation.

**Table 1:** Detection of fetuses with RAA and their lateral distribution of the ductus arteriosus.

Type	Cases *n* (%)	Lateral distribution of the ductus arteriosus			
		L	R	D	A
RAA-ALSA	78 (49.7%)	67 (85.9%)	8 (10.3%)	0 (0.0%)	3 (3.8%)
RAA-MB	75 (47.8%)	41 (54.7%)	27 (36.0%)	1 (1.3%)	6 (8.0%)
RAA-ILSA	3 (1.9%)	2 (66.7%)	0 (0.0%)	1 (33.3%)	0 (0.0%)
RAA-ILINA	1 (0.6%)	1 (100.0%)	0 (0.0%)	0 (0.0%)	0 (0.0%)
Total	157	111 (70.7%)	35 (22.3%)	2 (1.3%)	9 (5.7%)

L: left ductus arteriosus; R: right ductus arteriosus; D: double ductus arteriosus; A: absence of ductus arteriosus.

### The combined malformations of RAA

Among the 157 fetuses with RAA, 50 (31.85%) were isolated RAA, and 107 (68.15%) were nonisolated RAA, with the combined soft maker abnormality of 10.2% (16/157) and combined intracardiac and/or extracardiac abnormality of 58.0% (91/157). Among the combined soft makers, echogenic intracardiac focus and mild tricuspid regurgitation were predominant. With respect to intracardiac abnormalities, tetralogy of Fallot (TOF), ventricular septal defects and pulmonary atresia were more common, and the majority of extracardiac abnormalities were cleft lip and palate, entropion and hydronephrosis.

The incidence of combined abnormalities in the RAA-ILSA/ILINA group (collectively referred to as the isolated vessel group) was 100.0%, and the incidence of combined intracardiac and simultaneous intra- and extracardiac abnormalities was significantly greater in the RAA-MB group than in the RAA-ALSA group (Table 2). Among the DA types, the incidence of combined intracardiac anomalies was greater in the DDA, ADA, and RDA groups than in the LDA group, with statistically significant differences between the groups (Table 3). The most common associated congenital heart disease was TOF, with 19 cases, including 12 cases of LDA, 4 cases of RDA, 1 case of DDA, and 2 cases of ADA, followed by 8 cases of ventricular septal defect and 7 cases of pulmonary stenosis/atresia, 5 cases of right-sided anomalous syndrome, 3 cases of complete transposition of the great arteries, 1 case of perpetual trunk, 1 case of right heart dysplasia syndrome, 1 case of common right heart defect, and 1 case of common right heart defect. Dysplasia syndrome was present in 1 patient, common arterial trunk was present in 1 patient, and double outlet right ventricle was present in 1 patient. The combined intracardiac anomalies of the DDA and ADA groups were both TOF and right-sided heterotaxy syndrome.

**Table 2:** Comparison of associated abnormalities between fetuses with RAA-ALSA and RAA-MB.

	RAA-ALSA (*n* = 78)	RAA-MB (*n* = 75)	*P*
Associated abnormalities	41 (52.6%)	62 (82.7%)	0.001
Soft markers	10 (12.8%)	6 (8.0%)	0.330
Cardiac	15 (19.3%)	29 (38.7%)	0.008
Extracardiac	6 (7.7%)	0 (0.0%)	0.014
Cardiac and extracardiac	10 (12.8%)	27 (36.0%)	0.001

**Table 3:** Comparison of various types of DA in fetuses with RAA.

	Total (*n* = 157)	LDA (*n* = 111)	RDA (*n* = 35)	DDA (*n* = 2)	ADA (*n* = 9)	*P* value
Isolated RAA	50 (31.8%)	44 (36.9&)	6 (17.1%)	0 (0.0%)	0 (0.0%)	0.002
Nonisolated RAA	107 (68.2%)	67 (60.4%)	29 (82.9%)	2 (100.0%)	9 (100.0%)	0.002
Soft markers	16 (10.2%)	14 (12.6%)	2 (5.7%)	0 (0.0%)	0 (0.0%)	0.269
Cardiac abnormalities	46 (29.3%)	27 (24.4%)	14 (40.0%)	2 (100.0%)	3 (33.3%)	0.042
Extracardiac abnormalities	6 (3.8%)	6 (5.4%)	0 (0.0%)	0 (0.0%)	0 (0.0%)	0.235
Cardiac and extracardiac abnormalities	39 (24.9%)	20 (18.0%)	13 (37.2%)	0 (0.0%)	6 (66.7%)	0.003
Chromosomal abnormalities	8/62 (12.9%)	6/52 (11.5%)	2/8 (25.0%)	0/0 (0.0%)	0/2 (0.0%)	0.474

### Chromosomal aberrations in fetuses with RAA

The 62 cases with chromosome examinations included 34 cases of isolated RAA and 28 cases of nonisolated RAA. There was only 1 case of chromosomal abnormality in a fetus with isolated RAA, which manifested as a rosette anomaly. There were 7 cases of chromosomal abnormalities in fetuses with nonisolated RAA, specifically 3 cases of chromosomal microdeletions in fetuses with RAA-ALSA, including 1 case of 22q11.21 microdeletion, 1 case of 1q42.12q44 microdeletion, and 1 case of q15.1q15.3 microdeletion, and 4 cases of abnormalities in fetuses with RAA-MB, including 1 case of trisomy 21, 1 case of Turner syndrome, 1 case of 22q11.21 microdeletion and 1 case of 18q22.3q23 microduplication. The rate of chromosomal abnormalities in fetuses with nonisolated RAA (7/28, 25.0%) was greater than that in those with isolated RAA (2.9%, 1/34), and the difference between the two groups was statistically significant (*P* = 0.038). In addition, the differences in the rates of chromosomal abnormalities between the RAA-ALSA and RAA-MB groups were statistically significant (*P* = 0.05) (Table 4).

**Table 4:** Comparison of the incidence of chromosomal abnormalities between fetuses with RAA-ALSA and fetuses with RAA-MB.

Group	Tested cases	Cases (*n*, %)		*P* value
		Normal	Abnormal	
RAA-ALSA	43	40 (93.0%)	3 (7.0%)	0.05
Isolated	26	26 (100.0%)	0 (0.0%)	0.055
Nonisolated	17	14 (82.4%)	3 (17.6%)	
RAA-MB	19	14 (73.7%)	5 (26.3%)	
Isolated	8	7 (87.5%)	1 (12.5%)	0.338
Nonisolated	11	7 (63.6%)	4 (36.4%)	

### Pregnancy outcomes of fetuses with RAA

Among the cases of IUFD and intrauterine death in nonisolated RAA-MB, the causes were mainly due to the combination of severe intra- and extracardiac structural anomalies, of which 1 case was associated with chromosomal anomalies (Turner's syndrome). Both the RAA-ILSA and RAA-ILINA cases were combined with TOF, one of which was also combined with thymic dysplasia, and two live-born infants underwent postnatal surgical intervention and achieved good postoperative recovery. The remaining live-born infants were in good condition during follow-up, with no obvious tracheal or oesophageal compression symptoms.

Pregnancy outcomes varied between different groups, and a statistically significant comparison of the live birth rate was performed (*P* < 0.001). The live birth rate of the RAA-ALSA group (66.7%, 52/78) was significantly greater than that of the RAA-MB group (29.3%, 22/75). Furthermore, the live birth rate was 98.0% (49/50) in the isolated RAA group and 25.2% (27/107) in the nonisolated RAA group, with a statistically significant difference between the two groups (*P* < 0.0001) (Table 5).

**Table 5:** Comparison of the pregnancy outcomes of different groups of fetuses with RAA.

Type	Pregnancy outcome			*P* value
	Survival	TOP	IUFD	
RAA-ALSA	52 (66.7%)	26 (33.3%)	0 (0.0%)	<0.001
Isolated	36 (97.3%)	1 (2.7%)	0 (0.0%)	<0.001
Nonisolated	16 (39.0%)	25 (61.0%)	0 (0.0%)	
RAA-MB	22 (29.3%)	52 (69.3%)	1 (1.4%)	
Isolated	13 (100.0%)	0 (0.0%)	0 (0.0%)	<0.001
Nonisolated	9 (14.5%)	52 (83.9%)	1 (1.6%)	
RAA-ALSA	1 (33.3%)	2 (66.7%)	0 (0.0%)	1.000
RAA-ILINA	1 (100.0%)	0 (0.0%)	0 (0.0%)	1.000

TOP, termination of pregnancy; IUFD, intrauterine fetal demise.

### Diagnostic condition of prenatal ultrasound in RAA fetuses

Among the 4 (4/76, 5.3%) misdiagnosed cases, including 1 cases with RAA-MB with a postnatal echocardiogram suggestive of DAA and 3 cases with RAA-MB with postnatal echocardiograms suggestive of RAA-ALSA. Besides, 19 cases involved partial cardiac anatomy or vascular casting. The results revealed that the findings of 18 patients were consistent with the prenatal ultrasound findings, and those of one patient were inconsistent, resulting in a misdiagnosis of RAA combined with ventricular septal defect-type pulmonary atresia with RAA combined with a common truncus arteriosus.

## Discussion

With the continuous development of diagnostic techniques in fetal echocardiography and the progressive understanding of fetal RAA, the prenatal detection rate of RAA has increased. The detection rate of RAA in our study was 0.2%, which is similar to the prevalence of approximately 0.1% for RAA reported in previous literature (16). We demonstrated the value of prenatal ultrasound in the diagnosis of RAA by using prenatal ultrasound and case data, summarising the characteristic ultrasound manifestations, genetic features, and associated malformations of RAA and tracking the prognostic outcomes.

The main prenatal ultrasound manifestation of RAA is that the aortic arch located on the right side of the trachea, crossing the right principal bronchus and the right pulmonary artery. The pulmonary bifurcation view, three-vessel view, three-vessel-tracheal view, long-axis view of the aortic arch, coronary view of the descending aorta, and bilateral subclavian artery view play major roles in the diagnosis of RAA. Most cases need to be combined with sensitive and high-definition colour flow patterns for dynamic tracking in a forward or inverse direction to classify the type of RAA (3, 17). In this study, the most common type was RAA-ALSA, followed by RAA-MB, RAA-ILSA and RAA-ILINA. The accurate prenatal diagnosis of RAA-ILSA and RAA-ILINA is relatively difficult, and a focus on clarifying whether the subclavian or innominate arteries are connected to the pulmonary artery is crucial.

RAA is accompanied by the DA with varying numbers, laterality and connectivity, and the DA with the aorta and its branches form complete or incomplete “O”-shaped, “U”-shaped or “C”-shaped vascular loops surrounding the trachea, which could cause varying degrees of symptoms by compressing the trachea and/or oesophagus. Prenatal ultrasound detection of vascular rings has been performed mainly in three-vessel tracheal views and bilateral subclavian arterial views (18). In this study, the “U”-shaped incomplete vascular ring formed by RAA-ALSA with LDA and the “O”-shaped complete vascular ring formed by RAA-MB with DDA were more common. The increased diagnostic rate may be due to the routine detection of the bilateral subclavian arterial view and the application of the three-step ultrasound method to examine the aortic arch and its branches in our centre. In addition, the DA is an open physiological pathway in the fetal period, and there is no gas in the thoracic cavity interfering with the transmission of acoustic waves, which results in better clarity of images and makes the detection of vascular collaterals easier in this period than that in the postnatal period (19). To obtain an accurate diagnosis of RAA combined with a vascular ring, it is important to trace the course and bifurcation of the first branch of the aortic arch or the innominate artery and to explore the relationship between the vagus vessels and the aorta (20). In cases of combined TOF, the DA may be absent or originate from the subclavian artery, which makes it difficult to identify on fetal prenatal echocardiography and requires a combination of multiple views to assist in follow-up observation.

In addition, the pregnancy outcome of fetal RAA is closely related to the type and the severity of the combined anomalies (21, 22), and the prognosis of nonisolated RAA was significantly worse than that of isolated RAA in the present study. Among the types of RAA, RAA-MB had a high rate of combined intra- and extracardiac abnormalities, in the most common of which was TOF, and a relatively poor prognosis, whereas RAA-ALSA was less likely to be associated with malformations and generally had a better prognosis. Among the concomitant DA types, LDA with vertical alignment was usually combined with congenital heart disease, most commonly TOF (23). With regard to merged vascular rings, which constitute a relatively loose internal space within the “U”-shaped vascular ring, most fetuses are asymptomatic, with occasional feeding difficulties, and most have a good long-term prognosis without residual symptoms or late complications (24). None of the children in this study showed signs of oesophageal or tracheal obstruction after birth. Therefore, according to the anatomical characteristics of the different types of RAA and concomitant DA and vascular rings, useful prognostic influences can be provided for fetuses with RAA (25). When RAA is diagnosed prenatally, extracardiac anomalies should be excluded exhaustively, and if the type is RAA-MB, close monitoring of fetal outcome and prognostic assessment are important guidelines for clinical decision-making.

RAA has been reported to be associated with genetic abnormalities, with an incidence of chromosomal abnormalities of approximately 15.3%–24% according to Luciano D et al. (26). Among the cases in this study for which karyotype testing results were available, the incidence of chromosomal abnormalities in fetuses with isolated RAA was 2.9%, whereas that in fetuses with nonisolated RAA was as high as 25.0%, which implies that the risk of chromosomal abnormalities in fetuses with RAA associated with various abnormalities is significantly increased. The rate of combined chromosomal abnormalities in fetuses with RAA-MB (26.3%) was significantly higher than the rate in RAA-ALSA (7.0%) among the various types. Miranda et al. (27) reported that RAA-associated chromosomal abnormalities were more common with 22q11.2 microdeletion, with an incidence of up to approximately 20%. In our study, 22q11.2 microdeletion accounted for 25% (2/8) of the cases with chromosomal abnormalities.

Each type of fetal RAA has characteristic sonographic manifestations, and the comprehensive use of prenatal ultrasonography can diagnose and distinguish various types of RAA (28), but cases of misdiagnosis still occur. Previous studies have reported numerous reasons for misdiagnosis, including the technical skill of the operator, fetal orientation, fetal physiological channels and other circulatory conditions, abdominal wall transillumination window conditions, and amniotic fluid volume (29, 30). The reasons for misdiagnosis in our study may be as follows: (1) Instrument adjustment and operation problems: In this study, in one fetus with RAA-MB who underwent postnatal ultrasound, double aortic arch (DAA) was suggested, and related studies have shown that when there is distal dysplasia or atresia of the left aortic arch in the DAA, its ultrasound manifestation is extremely similar to that of RAA-MB, which makes it easy to misdiagnose the DAA as RAA-MB. The key point of differentiation between the two through our experience is whether the first branch is connected to the descending main body, which can be identified by combining multisections with comprehensive dynamic observations. (2) Lack of subjective awareness: In the present study, 3 fetuses with RAA-ALSA and one fetus with RAA-MB were inconsistently diagnosed due to a lack of awareness of the postnatal aortic arch and its branching pattern by the examiners. (3) Limited conditions of the foetus itself: Some fetuses have a variety of other serious intracardiac structural anomalies that make it difficult to visualise the branches of the aortic arch and the ductus arteriosus, which makes accurate prenatal diagnosis and differentiation challenging.

## Limitations

This was a single-centre study with a retrospective analysis, and data on long-term outcomes were unavailable. Only patients with verified diagnoses were included in this cohort. We have limited clinical follow-Up data. Not all fetuses with RAA had genetic testing results, and some lacked relevant pathological confirmation after termination of pregnancy and images postnatally. Future multicentre studies and further genetic verification are urgently needed.

## Conclusions

Prenatal ultrasonography has crucial value in diagnosing fetal RAA. 3VT views combined with coronal views of the trachea and its branches are essential for diagnosing RAA. The course of the first branch of the aortic arch or the innominate artery should be traced, and attention should be given to potential complications with other intracardiac malformations. The prognosis of fetal RAA is related to the severity of cardiac and extracardiac malformations and chromosomal abnormalities. Among the types of RAA, RAA-MB significantly differed from the other 3 types in terms of comorbid malformations, chromosomal abnormalities, and prognostic outcomes. When nonisolated RAA is detected, genetic testing is necessary to explore its correlation with genetic abnormalities. Pathological anatomy assessment and postnatal images may contribute to a better understanding of RAA. The main reasons for misdiagnosis by prenatal ultrasound include instrumental adjustment, a lack of subjective knowledge, fetal condition limitations, and disease progression. Attention should be given to follow-up management and validation of fetal RAA to provide a scientific basis for accurate prenatal diagnosis, clinical management, and decision-making research on RAA.

## Data Availability

The original contributions presented in the study are included in the article/Supplementary Material, further inquiries can be directed to the corresponding authors.
